# A Significant Increase in the Incidence of Central Precocious Puberty among Korean Girls from 2004 to 2010

**DOI:** 10.1371/journal.pone.0141844

**Published:** 2015-11-05

**Authors:** Shin Hye Kim, Kyoung Huh, Sungho Won, Kuk-Wha Lee, Mi-Jung Park

**Affiliations:** 1 Department of Pediatrics, Sanggye Paik Hospital, Inje University, College of Medicine, Seoul, Korea; 2 Kium Pediatric Clinic, Seoul, Korea; 3 Department of Public Health Science, Seoul National University, Seoul, Korea; 4 Division of Pediatric Endocrinology, Mattel Children's Hospital at University of California at Los Angeles, David Geffen School of Medicine, Los Angeles, California, United States of America; INIA, SPAIN

## Abstract

**Background:**

Few studies have explored the trends in central precocious puberty (CPP) in Asian populations. This study assessed the prevalence and annual incidence of CPP among Korean children.

**Methods:**

Using data from the Korean Health Insurance Review Agency from 2004 to 2010, we reviewed the records of 21,351 children, including those registered with a diagnosis of CPP for the first time and those diagnosed with CPP who were treated with gonadotropin-releasing hormone analogs.

**Results:**

The prevalence of CPP was 55.9 per 100,000 girls and 1.7 per 100,000 boys, respectively. The overall incidence of CPP was 15.3 per 100,000 girls, and 0.6 per 100,000 boys. The annual incidence of CPP in girls significantly increased from 3.3 to 50.4 per 100,000 girls; whereas in boys, it gradually increased from 0.3 to 1.2 per 100,000 boys. The annual incidence of CPP in girls consistently increased at all ages year by year, with greater increases at older ages (≥6 years of age), and smaller increases in girls aged < 6 years. In contrast, the annual incidence remained relatively constant in boys aged < 8 years, while a small increase was observed only in boys aged 8 years. The increase of annual incidence showed significant differences depending on age and gender (*P* <0.0001).

**Conclusions:**

The annual incidence of CPP has substantially increased among Korean girls over the past 7 years. Continued monitoring of CPP trends among Korean children will be informative.

## Introduction

Precocious puberty is generally defined by the onset of secondary sexual characteristics before the age of 8 yrs in girls and 9yrs in boys. This is classified as central or peripheral precocious puberty according to the primary source of the hormonal production [1]. If precocious puberty results from the premature maturation of hypothalamic-pituitary-gonadal axis, the condition is called central precocious puberty (CPP). CPP can cause early menarche in girls, loss of final height due to early epiphyseal fusion, and psychosocial problems especially in those with early onset and rapid progression [2]. Proper diagnosis and treatment can help to avoid these negative consequences. Diagnostic age limits were arbitrarily derived from studies on normal pubertal development in the general population, therefore, these age limits may change over time with secular trends. The mean age of pubertal onset in girls and boys has declined over the last 2 decades in countries including the United States of America [3], European nations [4,5], and China [6,7]. This secular trend of earlier occurrence of puberty is also apparent in Korea [8,9]. There is a possibility that the prevalence and incidence of CPP might have increased over the past decades, as pubertal timing has accelerated in the general population with no changes in the diagnostic criteria of CPP.

Additional epidemiologic data is needed to establish optimal guidelines for the diagnosis and treatment of CPP. In this report, we aimed to investigate the prevalence and incidence of CPP among Korean children, using data from a national registry.

## Methods

### Ethics statement

The survey data are publicly available, and patient records/information was anonymized and de-identified prior to analysis. This study was conducted according to the guidelines defined in the Declaration of Helsinki and all procedures involving human subjects were ethically approved by the Institutional Review Board at Inje University College of Medicine.

### Study Samples

All Korean people who visit hospitals are registered with the Korean Health Insurance Review Agency (HIRA), and their diagnoses are recorded for health insurance claims according to the *International Classification of Diseases*, *10th Revision* (ICD-10) coding system. Insurance claims for gonadotropin-releasing hormone analogs (GnRHa) are possible when a diagnosis of CPP is confirmed using the following criteria: the appearance of secondary sex characteristics defined as breast development in girls aged <8 years (Tanner stage 2 breast development, which is assessed via inspection and palpation), and testicular enlargement in boys aged <9 years (Tanner stage 2 genital development, determined as testicular volume >4 mL) combined with growth acceleration, the presence of advanced bone age, and documentation of a pubertal hormonal response defined as a peak luteinizing hormone of >5 IU/L after gonadotropin-releasing hormone (GnRH) stimulation testing [1]. We included boys aged <9 years and girls aged <8 years who visited hospitals for evaluations of CPP and registered with HIRA for the first time with an ICD-10 diagnosis of precocious puberty from 2004 to 2010. In addition, patients of both genders and of the same ages who had made insurance claims for GnRHa treatment based on a CPP diagnosis during the same study period were included in the investigation. New cases of CPP were defined as those children who claimed GnRHa treatment for the first time to HIRA.

### Calculations of Prevalence and Incidence

Data relating to the population at risk and specifically boys aged <9 years and girls aged <8 years from 2004 to 2010, were obtained from the National Institute of Statistics of Korea [10]. We calculated the prevalence of CPP as: (the number of total cases of CPP in 2010/population at risk in 2010) × 100,000. In addition, we calculated the annual incidence of CPP for each calendar year, age, and gender as: (the number of new cases of CPP in the year studied/population at risk in that year) × 100,000. We assumed that the number of CPP follows the Poisson distribution, and 95% confidence intervals of incidence estimates were obtained from the asymptotic convergence of incidence estimates to the normal distribution.

### Generalized Linear Mixed Models

The numbers of CPP in each year were obtained for each birth cohort, and we conducted statistical analyses to confirm whether there are significant differences according to age and gender. The numbers of CPP can be assumed to follow the Poisson distribution and thus we considered the generalized linear mixed model. The numbers of CPP in each birth cohort are expected to be similar and effects of birth cohort were considered as a random effect. Age, age^2^, gender and age × genders were included as fixed effects in our generalized linear mixed model. The incidence of CPP for each birth cohort were considered as an offset and we utilized the canonical link function. We utilized the Laplace method for numerical approximation and coefficients of fixed effects were estimated with glimmix procedure in SAS version 9.3.

## Results

### Patients with Central Precocious Puberty

The numbers of cases who were medically examined for the first time for CPP and those who were treated with GnRHa on the basis of a diagnosis of CPP are shown in Table 1. Of children suspected of having CPP, 10.4% of girls and 8.3% of boys were confirmed as having CPP and underwent GnRHa treatment. The numbers of children of both genders who were evaluated for precocious puberty consistently increased from 2004 to 2010. The percentage of patients diagnosed with CPP has increased since 2008, particularly in girls.

**Table 1:** The percentage of children treated with gonadotropin-releasing hormone analogs in children evaluated for central precocious puberty.

	Total			Boys			Girls		
Year	Evaluated for CPP (n)	CPP (n)	Percentage of children treated with GnRHa (%)	Evaluated for CPP (n)	CPP (n)	Percentage of children treated with GnRHa (%)	Evaluated for CPP (n)	CPP (n)	Percentage of children treated with GnRHa (%)
2004	1,093	82	7.5	75	9	12.0	1,018	73	7.3
2005	1,579	108	6.8	99	11	11.1	1,480	97	6.5
2006	1,709	97	5.7	107	9	8.4	1,602	88	5.5
2007	2,603	172	6.6	157	12	7.6	2,446	160	6.5
2008	3,620	340	9.3	157	13	8.3	3,463	327	9.4
2009	4,901	474	9.6	271	15	5.5	4,630	459	9.9
2010	5,846	923	15.8	273	25	9.1	5,573	898	16.1
**Total**	**21351**	**2,196**	**10.3**	**1,139**	**94**	**8.3**	**20212**	**2,102**	**10.4**

### The Prevalence of Central Precocious Puberty

The prevalence of CPP in 2010 is provided in Table 2. The prevalence of CPP was 55.9 per 100,000 children in girls and 1.7 per 100,000 children in boys.

**Table 2:** The prevalence of central precocious puberty in 2010.

	Total	Boys	Girls
Patients, n	1,033	37	996
Population at risk	3,963,320	2,180,672	1,782,648
Prevalence of CPP (patients per 100,000 children)	26.1	1.7	55.9

### The Incidence of Central Precocious Puberty

**Fig 1:**
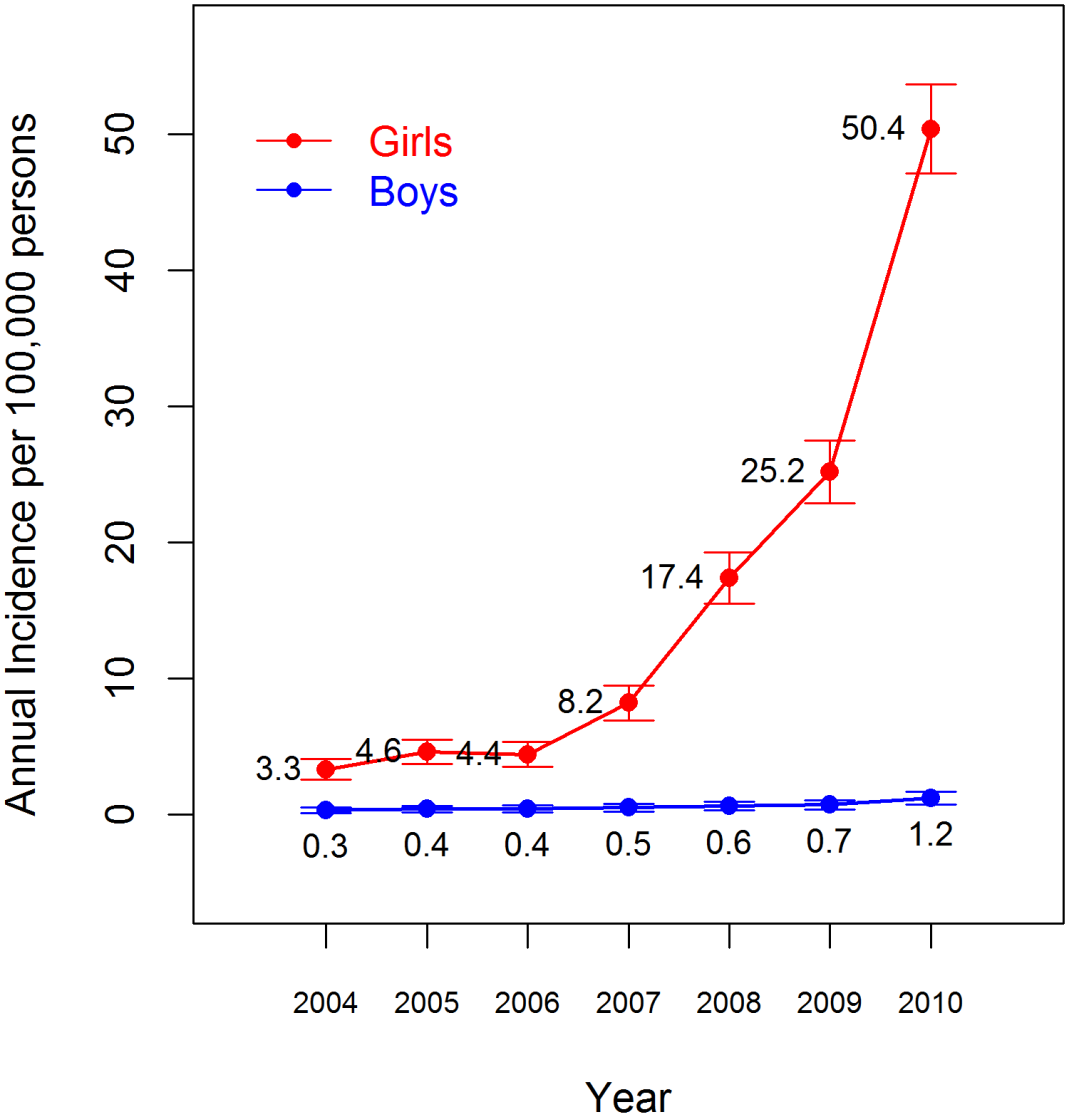
The annual incidence of central precocious puberty among Korean children. The error bars represents the 95% confidence intervals of the incidence estimates.

The annual incidence of CPP according to gender and the year of diagnosis is shown in Table 3 and Fig 1. The annual incidence of CPP in girls significantly increased from 3.3 to 50.4 per 100,000 girls during the study period. In boys, the annual incidence of CPP increased gradually from 0.3 to 1.2 per 100,000 boys. Girls showed an overall CPP incidence of 15.3 per 100,000 children over the time period studied. Girls were 25-times more likely to develop CPP than boys who had an overall incidence of 0.6 per 100,000 children.

**Table 3:** The annual and overall incidence of central precocious puberty.

	Total			Boys			Girls		
Year	Patients (n)	Population at risk (n)	Incidence	Patients (n)	Population at risk (n)	Incidence	Patients (n)	Population at risk (n)	Incidence
2004	82	5,010,225	1.6	9	2,791,694	0.3	73	2,218,531	3.3
2005	108	4,774,993	2.3	11	2,657,777	0.4	97	2,117,216	4.6
2006	97	4,545,226	2.1	9	2,528,053	0.4	88	2,017,173	4.4
2007	172	4,368,839	3.9	12	2,426,258	0.5	160	1,942,581	8.2
2008	340	4,234,864	8.0	13	2,351,580	0.6	327	1,883,284	17.4
2009	474	4,076,008	11.6	15	2,257,633	0.7	459	1,818,375	25.2
2010	923	3,963,320	23.3	25	2,180,672	1.2	898	1,782,648	50.4
**Total**	**2,196**	**30,973,475**	**7.1**	**94**	**17,193,667**	**0.6**	**2102**	**13,779,808**	**15.3**

Table 4 presents the annual incidence of CPP according to gender, the age at diagnosis and the year of diagnosis. Data are expressed as numbers of patients and (incidence per 100,000 persons). The annual incidence of CPP increased every year after 2007 in girls of all ages. Greater increases were observed at ages ≥ 6 years of age, and smaller increases were noted at ages < 6 years. In boys aged < 8 years, the annual incidence was constant during the study period, and gradually increased year by year in boys aged 8 years.

**Table 4:** The annual incidence of central precocious puberty according to gender, age, and year.

	Year							
	2004	2005	2006	2007	2008	2009	2010	2004–2010
Boys								
Age								
<6 y	2 (0.1)	2 (0.1)	1 (0.1)	1 (0.1)	1 (0.1)	2 (0.1)	1 (0.1)	10 (0.1)
<7 y	2 (0.1)	3 (0.2)	2 (0.1)	2 (0.1)	3 (0.2)	5 (0.3)	4 (0.2)	21 (0.2)
<8 y	5 (0.2)	5 (0.2)	5 (0.2)	6 (0.3)	5 (0.3)	8 (0.4)	10 (0.5)	44 (0.3)
<9 y	9 (0.3)	11 (0.4)	9 (0.4)	12 (0.5)	13 (0.6)	15 (0.7)	25 (1.2)	94 (0.6)
Girls								
Age								
<6 y	12 (0.8)	9 (0.6)	7 (0.5)	13 (1.0)	13 (1.0)	16 (1.2)	36 (2.7)	106 (1.1)
<7 y	32 (1.6)	29 (1.6)	24 (1.4)	44 (2.7)	73 (4.6)	85 (5.4)	193 (12.5)	479 (4.1)
<8 y	73 (3.3)	97 (4.6)	88 (4.4)	160 (8.2)	327 (17.4)	459 (25.2)	898 (50.4)	2,102 (15.3)

Table 5 shows the estimated differences of CPP incidence according to age and gender using a generalized mixed model. The effects of age, age^2^, gender and age×gender are significantly different at the 0.05 significance level. Therefore, we concluded that the incidence of CPP depends on the age and gender, and the annual increment of incidence significantly differs between boys and girls.

**Table 5:** Estimated differences of CPP incidence among Korean children according to age and gender.

	Estimate	Standard error	Z statistic	P-value
Age	3.373	0.151	22.31	<0.0001
Age^2^	-0.620	0.062	-10.06	<0.0001
Gender	-3.243	0.312	-10.39	<0.0001
Age×gender	-0.301	0.149	-2.01	0.044

## Discussion

In this study, we demonstrated that the overall prevalence of CPP among Korean children in 2010 was 26.1 per 100,000 children, with a prevalence of 55.9 per 100,000 girls and 1.7 per 100,000 boys. Furthermore, from 2004 to 2010, the annual incidence of CPP rose steeply, in particular, among girls. This is the first epidemiologic study utilizing a large-scale national registry that has explored the prevalence and incidence of CPP in Asia.

Few reports have described the prevalence of precocious puberty. The first report about the prevalence of precocious puberty that was based on nationwide data was from Denmark in 2005, and the estimate of precocious puberty incidence in girls younger than 9 years was 200 per 100,000 person years and it was 50 per 100,000 person years in boys who were younger than 10 years [11]. However, this study included patients with true CPP, premature thelarche, premature adrenarche, which might lead to overestimation; moreover, the age limit for CPP was extended by about 1 yr. Another reports from Spain [12] estimated that the overall prevalence of CPP was 19 per 100,000 persons, and that the prevalences for both genders were 37 per 100,000 girls and 0.46 in 100,000 boys. However, these data were gathered from 90% of tertiary care centers with pediatric endocrinology, and this may have caused underestimation of the number of patients. In the current study, the CPP prevalence among Korean girls was 55.9 per 100,000 girls, which is 1.5-fold higher than that observed with Spanish girls, and the CPP prevalence among Korean boys was 1.7 per 100,000 boys, which appears to be 3.7-fold higher than that observed with Spanish boys [12].

Studies on secular trends in precocious puberty are also very limited in number. A Danish study showed that from 1993 to 2001, the annual incidence of precocious puberty was constant between 15 and 29 per 100,000 girls and < 0.5 per 100,000 boys [11]. The incidence estimates of CPP within the Spanish population between 1997 and 2009 ranged from 0.13 to 2.17 per 100,000 children in girls and 0 to 0.23 per 100,000 children in boys [12]. An increase in the annual incidence of CPP among Spanish children was evident since the first cases of CPP in adopted children were reported in 2000 [12]. For adopted children, the annual incidence of CPP was 27-fold higher than that of Spanish children overall, and it ranged from 14–62 per 100,000 girls and 0–15 per 100,000 boys [12]. In the present study, we demonstrate that the annual incidence of CPP increased from 3.3 to 50.4 per 100,000 girls and from 0.3 to 1.2 per 100,000 boys between 2004 and 2010. The annual incidence of CPP in Korea, which was comparable to that of Spanish children between 2004 and 2006, consistently increased since 2007 and reached the incidence rates that had been reported in Spanish adoptees by 2010.

It is unclear whether the steep rise in Korea’s CPP incidence is the result of an actual increase in the number of CPP patients, because such a large increase of a disease over this relatively short period is uncommon. A shift in the entire population towards earlier pubertal onset would be a possible explanation. We previously demonstrated that age of menarche has been advanced approximately by 2 years between 1900s and 1980s birth cohorts, and 1980s birth cohorts had 11 times higher odds for early menarche (experiencing menarche 2 years earlier than average) than 1900s birth cohorts [9]. Our finding of an increasing incidence of CPP during 2004–2010 might reflect an ongoing trend towards earlier activation of the HPG axis in Korean children who were born during 1997–2003. On the other hand, recent studies on pubertal development among North American and Danish children have indicated that the onset of breast development is occurring one year earlier than 15–20 years ago, but that age of menarche has declined to a lesser extent (3 months) during the same period [4,13]. The Danish study suggested that this trend might be independent of the activation of the hypothalamic-pituitary-gonadal (HPG) axis, because no differences were observed between 2 cohorts that were studied 15 years apart in relation to the gonadotropin levels [4]. Recent studies indicates that higher body mass index and the percentage of body fat independently predict earlier menarche in Korean girls [14,15]. Leptin, a hormone secreted by adipocytes, not only directly stimulates the release of gonadotropin from the anterior pituitary gland, but it also stimulates the hypothalamic ventral premammillary neurons to release glutamate, which activates GnRH neurons [16]. Since the prevalence of obesity among Korean children and adolescents also rose rapidly by about 2-folds between 1997 and 2005 [17], this observation may partly explain a shift of the Korean girls towards earlier activation of the HPG axis resulting in increase in CPP. Another possible reason for this finding is an increase in parental awareness for CPP leading to a rise in treatment. As a consequence, cases with slowly progressive or non-sustained CPP who do not need treatment might have been treated with GnRHa possibly by parental insistence on treatment or by prescription of inexperienced physicians. It is noteworthy that incidence in boys and in girls aged <6 years (which may represent a clear pathologic condition) showed only a modest increase during the study period.

There were some limitations in our study. Since we could not assess detailed medical information about the patients, we were unable to evaluate factors potentially involved in the increase in CPP in Korea. Nonetheless, this is the first study that has determined recent CPP prevalence and annual incidence in an Asian country that are based on the data from a national registry.

In conclusion, this study demonstrates a significant increase in the incidence of CPP among Korean children over the period of 2004–2010, particularly among girls. We propose that the incidence of CPP and potential factors associated with the risk of CCP must be monitored in Korean children during the course of future decades.
